# Supplementary material for: Intake of Processed Meat and Association with Sociodemographic and Lifestyle Factors in a Representative Sample of the Swiss Population
Source: Nutrients. 2019 Oct 23;11(11):2556. doi: 10.3390/nu11112556 (PMC6893731; doi:10.3390/nu11112556)
Supplement: Supplementary file 1 [file nutrients-11-02556-s001.pdf]

Supplementary Materials:

Intake of processed meat and associations with sociodemographic and lifestyle factors in a representative sample of the Swiss population.

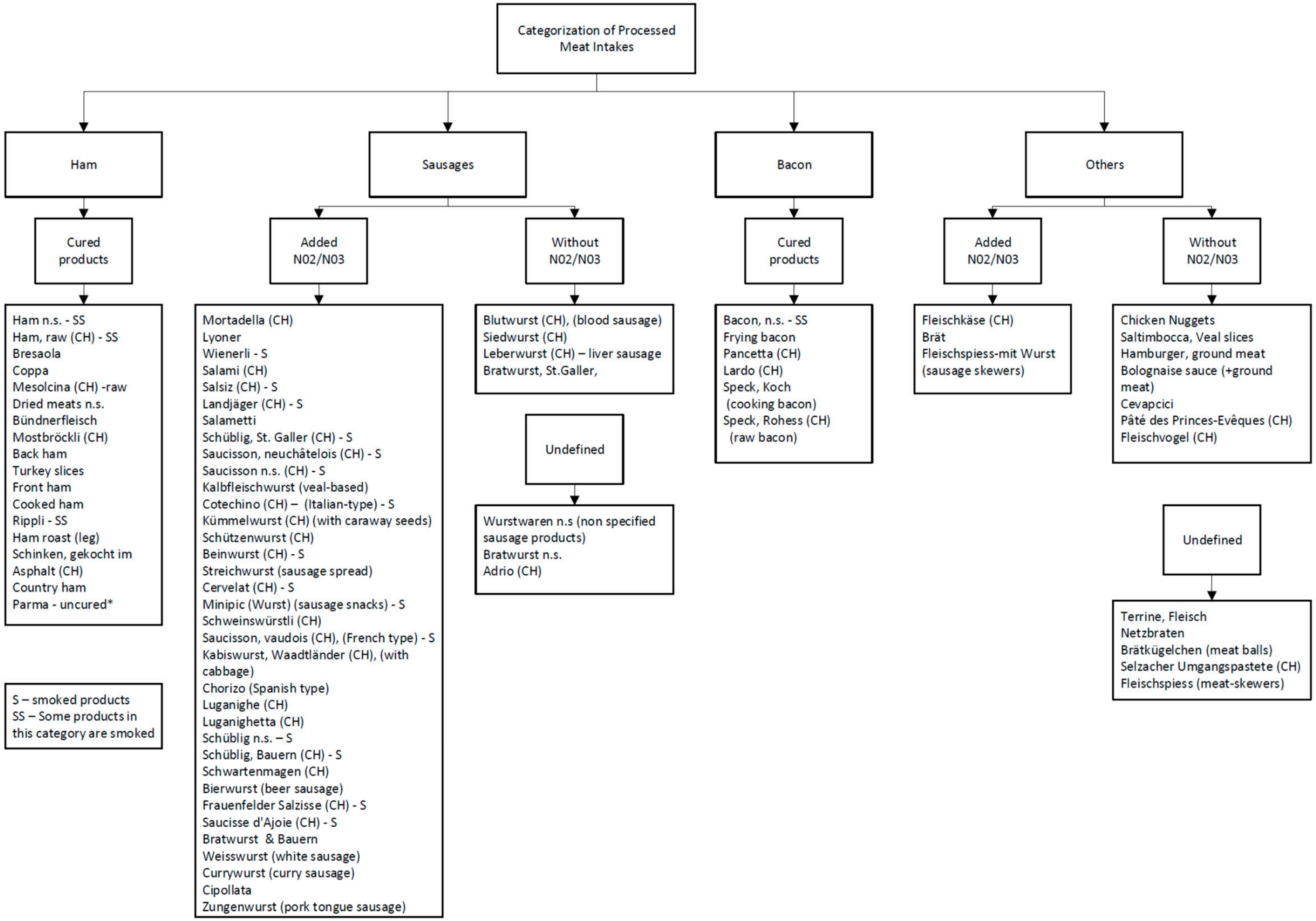


**Figure S1.** Categorization of processed meat consumptions, National Nutrition Survey menuCH, N=2,057.

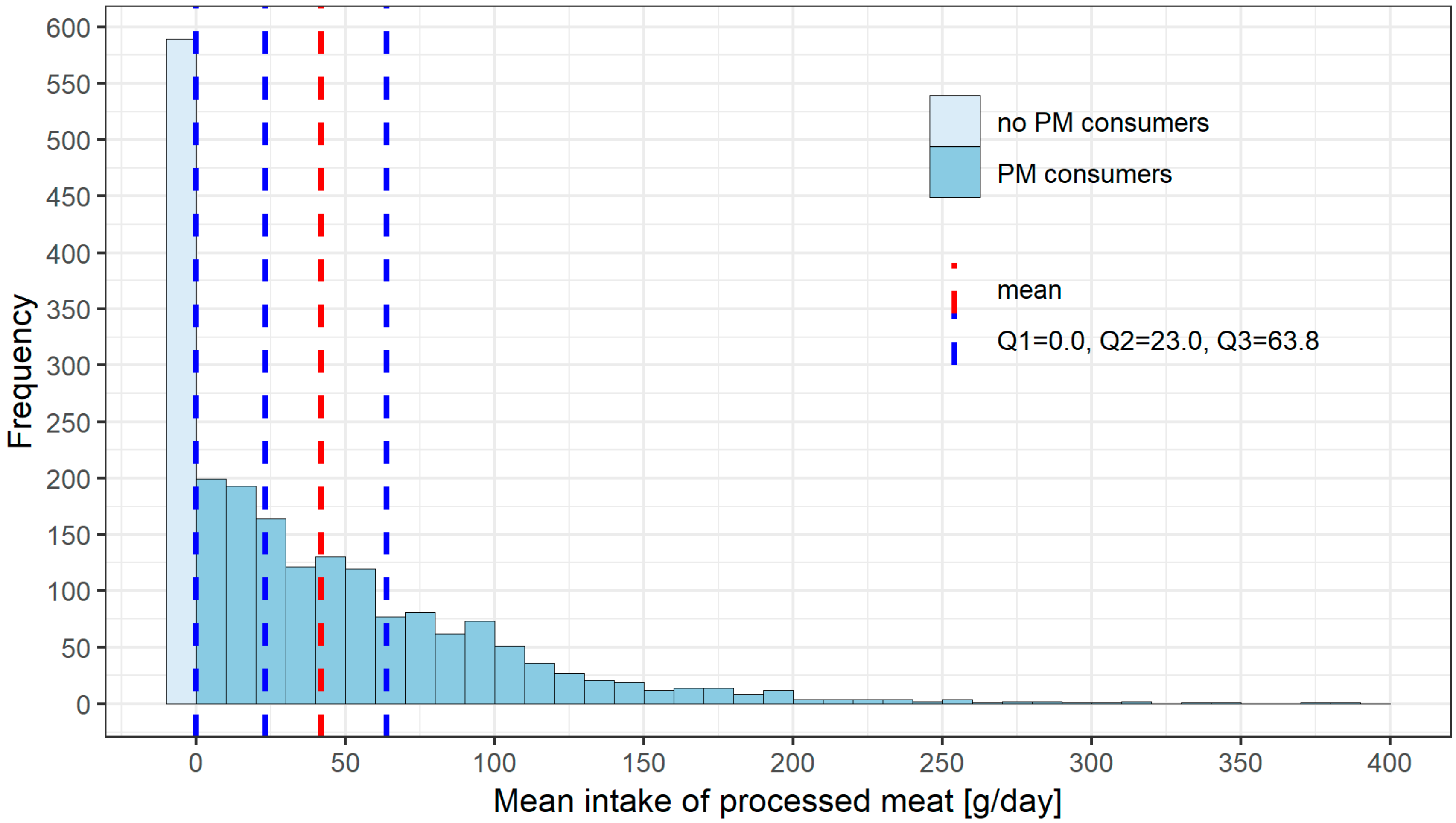


**Figure S2.** Histogram of processed meat (PM) intakes by the population, National Nutrition Survey menuCH $N$ = 2,057 crude data.
